# Molecular Detection of Integrons, Colistin and *β-lactamase* Resistant Genes in *Salmonella enterica* Serovars Enteritidis and Typhimurium Isolated from Chickens and Rats Inhabiting Poultry Farms

**DOI:** 10.3390/microorganisms10020313

**Published:** 2022-01-28

**Authors:** Tsepo Ramatla, Kealeboga Mileng, Rendani Ndou, Nthabiseng Mphuti, Michelo Syakalima, Kgaugelo E. Lekota, Oriel M.M. Thekisoe

**Affiliations:** 1 Department of Animal Health, School of Agriculture, North-West University, Private Bag X2046, Mmabatho 2735, South Africa; k.mileng@gmail.com (K.M.); Rendani.Ndou@nwu.ac.za (R.N.); nthabiseng.mphuthi@nwu.ac.za (N.M.); michsan65@gmail.com (M.S.); 2 Unit for Environmental Sciences and Management, North-West University, Private Bag X6001, Potchefstroom 2531, South Africa; Lekota.Lekota@nwu.ac.za (K.E.L.); thekisoe@gmail.com (O.M.M.T.); 3 Department of Disease Control, School of Veterinary Medicine, University of Zambia, Lusaka P.O. Box 32379, Zambia

**Keywords:** integrons, colistin, *β-lactamase*, *Salmonella* serovars, antibiotic resistance

## Abstract

The rapid growth of multidrug-resistant *Salmonella* is a global public health concern. The aim of this study was to detect integrons, colistin and *β-lactamase* resistance genes in *Salmonella enteritidis* and *typhimurium*. A total of 63 isolates of *S. enteritidis* (*n* = 18) and *S. typhimurium* (*n* = 45) from fecal samples of layers and rats at chicken farms were screened for antibiotic resistant genes. Conventional PCR was performed for the detection of integrons (classes 1, 2, and 3), colistin (*mcr*-1-5) and β-lactamase (*bla_CTX-M_*, *bla_CTX-M-1_*, *bla_CTX-M-2_*, *bla_CTX-M-9_*, *bla_CTX-M_*_-15_, *bla_TEM_*, *bla_SHV_*, and *bla_OXA_*) resistant genes. Of these isolates, 77% and 27% of *S. typhimurium* and *S. enteritidis* harboured the *mcr-*4 encoded gene for colistin, respectively. The prevalence of class 1 integrons for *S. typhimurium* and *S. enteritidis* was 100% for each serovar, while for class 2 integrons of *S. typhimurium* and *S. enteritidis* it was 49% and 33% respectively, while class 3 integron genes was not detected. Our study also detected high levels of *β-lactamase* encoding genes (*bla* gene), namely *bla_CTX-M_*, *bla_CTX-M-1_*, *bla_CTX-M_*_-9_ and *bla_TEM_* from both *S. typhimurium* and *S. enteritidis*. This, to our knowledge, is the first report of *mcr-4* resistance gene detection in *Salmonella* serovars in South Africa. This study also highlights the importance of controlling rats at poultry farms in order to reduce the risk of transmission of antibiotic resistance to chickens and eventually to humans.

## 1. Introduction

*Salmonella* species are Gram-negative bacterial pathogens that are mostly associated with food poisoning outbreaks worldwide [1]. *Salmonella* serovars that cause human infection have been found to be more prevalent in chickens than in other animal types [2]. Contaminated poultry food products have been reported to be a source of more than 95% of non-typhoidal *Salmonella* (NTS) infections [3,4]. Shonhiwa et al. [5] mentioned that outbreaks of food-borne diseases (FBDs) reported in South Africa between January 2013–December 2017 resulted in 11,155 individual infections, with 78% hospital visits, 4% hospital admissions and 0.4% deaths. A majority of the outbreaks were recorded from KwaZulu-Natal (43%), Gauteng (19%), and Mpumalanga (12%) provinces during the warmer months. 

Antimicrobial resistance (AMR) is a growing global public health concern for humans and animals [6]. Several studies have revealed that antimicrobial use in food animals is a major contributor to the development of decreased susceptibility to antimicrobial agents in humans [7]. Microorganisms that are exposed to various antibiotics express resistance genes for protection and are capable of spreading their resistance genes to other non-pathogenic bacteria, thus creating resistance gene sources/reservoirs [8]. Antimicrobial resistance genes from food supplies have sparked increased interest in the public health sector [9]. 

Resistance to β-lactams, especially in Gram-negative bacteria, is primarily due to β-lactamase expression [10]. The β-lactams are widely used to treat infections in both animals and humans, especially infections which are due to *Salmonella* serovars [11]. *Salmonella* serovars are known to harbour the *bla_CTX-M_*, *bla_OXA_*, *bla_PER_*, *bla_SHV_*, *bla_TEM_*, *bla_CTX_*, and *bla_CMY_* genes that encode extended spectrum beta-lactamases (ESBL) resistance [11,12]. A study conducted in Denmark revealed that, among β-lactamase resistance genes, *bla_TEM-1b_* was the mostly detected gene [13]. However, *Salmonella* strains have a lower prevalence of ESBLs than other Gram-negative bacteria [14].

Antibiotic resistance against colistin (COL), is becoming more common and a point of concern because it is a last-resort antibiotic used against difficult-to-treat pathogens such as *Acinetobacter baumanni*, *Klebsiella pneumoniae*, and *Pseudomonas aeruginosa* [15,16,17,18,19,20]. Although COL use in humans is uncommon in Africa, it is widely used in livestock [16,19]. COL is an over-the-counter drug supplied and dispensed by non-professionals in most African nations apart from South Africa [19]. In 2016, the first plasmid-mediated colistin resistance gene, *mcr*-1, was discovered in animals and humans [15]. Besides being detected in either animals or humans, the *mcr* gene was originally discovered in seawater [21]. 

Integrons are defined by the presence of an integrase gene (*IntI*) [22]. They are genetic components that capture mobile gene cassettes that typically encode antimicrobial resistance determinants [23] and have been reported to contain one or more genes that code for antibiotic resistance [24]. The integrons are not considered as mobile genetic elements, although they can be transferred between bacteria by transposons or plasmids in which they are present [22,25]. About three types of integrons have been identified (*IntI*1, *IntI*2, and *IntI*3) [25]. The basic structure of integrons is composed of 5′ and 3′-conserved segments with gene cassettes containing antibiotic resistance genes [26]. They (*IntI*1, 2 and 3) have a primary recombination site (attI), a gene encoding an integrase belonging to the tyrosine-recombinase family (*intI*), and the 5′-conserved region contains a promoter (Pc) (22). Most genes that are responsible for *Salmonella* resistance have been found in class 1 integrons [27]. By using site-specific recombination, Class I integrons can incorporate AMR genes from the environment [28] and have been reported in many Gram-negative bacteria [25]. The Class 2 integrons are embedded in the Tn7 family of transposons and were reported in *Salmonella*, *Escherichia*, *Shigella* species and other isolates [25]. 

Effective antimicrobial therapy is crucial in the treatment of protracted salmonellosis [29]. According to Du et al. [30], erythromycin and ciprofloxacin are the most commonly used antimicrobial agents in clinics. The rising prevalence of multidrug resistance (MDR) by *Salmonella* spp. to clinically significant antimicrobial drugs such as β-lactams is currently an emerging concern because MDR bacteria can infect humans through the food supply [30,31]. The fundamental issue with resistant bacteria is the scarcity of antibiotics available for their treatment [32].

The aim of this study was to determine the occurrence and spread of the integron types, colistin and *β-lactamase* resistance genes in *Salmonella enterica* serovars Typhimurium and Enteritidis isolates recovered from chickens (layers) and rats at chicken farms in North West province of South Africa.

## 2. Materials and Methods

### 2.1. Salmonella serovars

A total of 274 fecal samples were collected from chickens (layers) (*n* = 120) and rats (*n* = 154) in six commercial farms, as described in our previous study [8]. The capturing of rats and identification was described in our previous study, Ramatla et al. [33]. *Salmonella* species were isolated from the feces by following the International Organization for Standardization method (ISO6579: 2002). Genomic DNA extraction using the Fungal/Bacterial Soil Microbe DNA Mini Prep kit, (Zymo Research, Irvine, CA, USA), PCR and sequencing were also carried out. All the sequenced isolates were deposited into the GenBank database and were assigned accession numbers. A total of 63 isolates of *Salmonella enteritidis* (*n* = 18) and *typhimurium* (*n* = 45) isolates were ultimately identified and used in this study.

### 2.2. Antibiotic Susceptibility Testing

The antibiotic resistance profile of the *Salmonella* serovars was determined using the Kirby-Bauer disc diffusion method on Mueller–Hinton agar [33]. The antibiotic panel consisted of 11 antibiotic discs (Davies Diagnostics, Johannesburg, South Africa) that included Sulphonamides (300 μg), Streptomycin (10 μg), Ampicillin (10 μg), Enrofloxacin (5 μg), Tetracycline (30 μg), Gentamicin (10 μg), Ciprofloxacin (5 μg), Rifampicin (μg), Chloramphenicol (30 μg), Nalidixic acid (30 μg) and Cephalothin (30 μg). The *E. coli* ATCC 25922 and *S. typhimurium* ATCC 14028 were used as negative and positive controls respectively. 

### 2.3. Detection of Antibiotic Resistance Genes

The isolates were screened for colistin genes (*mcr-1, mcr-2, mcr-3, mcr-4* and *mcr-5*) and *β-lactamase* (*bla_CTX-M_*, *bla_CTX-M-_*_1_, *bla_CTX-M-_*_2_, *bla_CTX-M_*_-9_, *bla_CTX-M_*_-15_, *bla_TEM__,_ bla_SHV_*, and *bla_OXA_*) resistance genes as well as different types of integrons (Class 1, 2 and 3). A molecular weight marker of 100 bp ladder (PROMEGA, Madison, WI, USA) was used to determine the size of the PCR amplicons.

### 2.4. Analysis of Antimicrobial Resistance Genes

#### 2.4.1. Detection of Colistin (*mcr*)

The fragments of the five *mcr* genes were amplified using a multiplex PCR, and the PCR conditions are presented in Table 1. The amplicon sizes of the *mcr*-1 to 5 ranged from 320 bp–1644 bp, respectively [34] (Table 1). Each PCR reaction was conducted in a total reaction volume of 25 μL containing 12.5 µL of the 2X DreamTag Green Master Mix (0.4 mM dATP, 0.4 mM dCTP 0.4 mM dGTP and 0.4 mM dTTP, 4 Mm MgCl2 and loading buffer), 8.5 µL of nuclease-free water, 1 µL of each oligonucleotide primer, and 1 µL of DNA template. Amplified PCR products were electrophoresed on a 1.5% (*w*/*v*) agarose gel stained with ethidium bromide and visualized under ultraviolet (UV) light. 

#### 2.4.2. Detection of *β-lactamase* Genes

All isolates were subjected to PCR amplification for detection of the *β-lactamase* resistance-encoding genes using primers listed in Table 1. The following genes encoding the *β-lactamase* mechanism [35] were investigated: *bla_CTX-M_*, *bla_CTX-M-_*_1_, *bla_CTX-M_*_-2_, *bla_CTX-M_*_-9_, *bla_CTX-M_*_-15_, *bla_TEM_*, *bla_SHV_*, and *bla_OXA_*. The PCR reaction consisted of the 2X DreamTaq Green Master Mix as mentioned above with PCR conditions shown in Table 1. 

#### 2.4.3. Detection of Integrons (*IntI*) Genes

The presence of *Int* (*IntI*1, *IntI*2, and *IntI*3) gene-encoding class 1 integrons was screened in all *S. enteritidis*, and *S. typhimurium* isolates using PCR. The primers listed in Table 1, were used to amplify the *Int* resistance genes [25,36,37]. The PCR reactions consisted of the 2X DreamTaq Green Master Mix as described above using PCR conditions as described in Table 1. 

## 3. Results

### 3.1. Antimicrobial Susceptibility Testing

**Figure 1:**
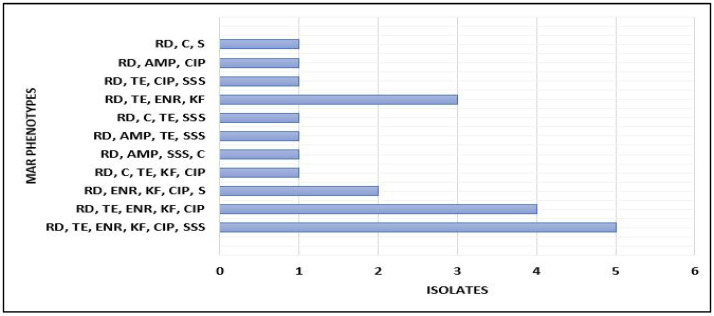
Multiple Antibiotic-Resistant Phenotypes pattern of *S. enteritidis* and *typhimurium* isolates. CA = Gentamicin, C = Chloramphenicol, CIP = Ciprofloxacin, RD = Rifampicin, NA = Nalidixic acid, AMP = Ampicillin, ENR = Enrofloxacin, TE = Tetracycline, KF = Cephalothin and SSS = Sulphonamide, S = Streptomycin.

All 63 *Salmonella enteritidis* and *typhimurium* isolates used in this study were resistant against enrofloxacin 61.9% (39/63), tetracycline 46.0% (29/63), streptomycin 33.3% (21/63), cephalothin 22.2% (14/63), sulphonamide 20.6% (13/63), gentamicin 17.5% (11/63), nalidixic acid 14.3% (9/63), rifampicin 9.5% (6/63), ampicillin 4.8% (3/63) and ciprofloxacin 3.2% (2/63). None of the isolates were resistant to chloramphenicol (Table 2). Figure 1 shows isolates that were multidrug-resistant. About 21 isolates showed resistance to at least three classes of antibiotics, with five isolates showing resistance to up to six out of 11 tested antibiotics.

### 3.2. Detection of Antibiotic Resistance Genes

The study revealed the presence of COL and *β-lactamase* antibiotic-resistant *S. enteritidis* and *S. typhimurium* isolates and as well as integrons. The gene encoding resistance to COL (*mcr-*4) was detected from 31 (49%) *Salmonella* isolates in this study. About 58% and 28% were detected from *S. typhimurium* and *S. enteritidis* isolates, respectively. Figures S1–S10 depict representative agarose gels containing PCR amplicons of the antibiotic resistance genes detected from this study.

**Figure 2:**
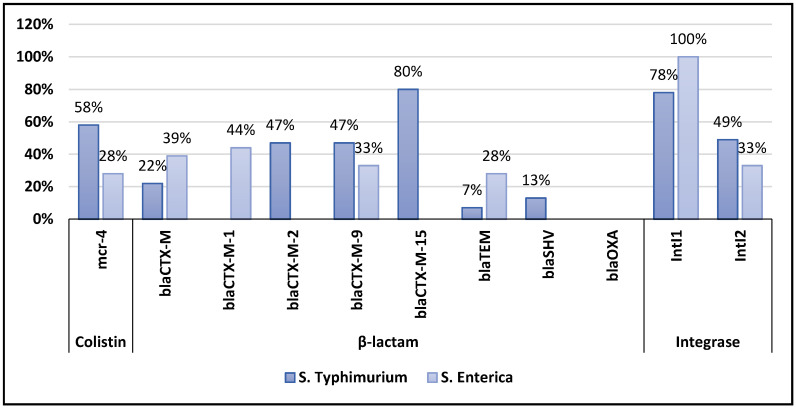
Distribution of antibiotic resistance genes among *Salmonella enteritidis* and *typhimurium* isolates.

In general, most of the isolates harboured *β-lactamase* encoding genes. A majority of *S. typhimurium* isolates consisted of ESBL encoding genes, including *bla_CTX-M_*_-9_, *bla_CTX-M_*_-2_, *bla_CTX-M_*_-15_, *bla_TEM_*, *bla_SHV_*, and *bla_CTX-M_* at 21 (47%), 21 (47%), 36 (80%), 3 (7%), 6 (13%) and 10 (22%), respectively. The summary of *bla* genes encoding *β*-lactam are shown in Figure 2. The bulk of *S. enteritidis* isolates carried *bla_CTX-M_*, *bla_CTX-M-_*_1_, *bla_CTX-M_*_-9_, and *bla_TEM_* genes at *n* = 7 (39%), *n* = 8(44%), *n* = 6 (33%), and *n* = 5 (28%) respectively, all encoding for resistance to *β-lactamase*, as shown in Table 3.

Out of 63 isolates, only 84% were harbouring *IntI*1 gene encoding class 1 integrons, of which 78% and 100% were detected in *S. typhimurium* and *S. enteritidis* isolates respectively. *IntI*2 genes encoding class 2 integrons were detected in 22 (49%) and 6 (33%), *S. typhimurium* and *S. enteritidis* isolates, respectively (Table 3). 

## 4. Discussion

Antibiotic resistance in *Salmonella* species has now become a global public health concern. In this study, the disc diffusion test was used to determine the antibiotic-resistant profiles in *Salmonella enterica* serovars Enteriditis and Typhimurium. Our results demonstrated high phenotypic resistance for enrofloxacin (61.9%), tetracycline (46.0%), and streptomycin (33.3%); however, a low antibiotic resistance was observed for ciprofloxacin (3.2%). Some of antimicrobial agents such as streptomycin ampicillin, chloramphenicol, gentamycin, and cefotaxime are not commonly used in animal health and production in South Africa [38]. In the current study, 21 (33.3%) of the isolates were multidrug-resistant. Our results are consistent with the findings of the previous studies conducted in Italy, Ghana and elsewhere in South Africa which reported multidrug resistance of 15%, 81.8% and 66.7% by *Salmonella* isolates, respectively [39,40,41].

The presence of integrons, colistin and *β-lactamase* resistant genes in *Salmonella* serovars continues to be a major food and public health burden worldwide, especially in poultry farming. Furthermore, detection of these resistant genes in rats around poultry houses highlights how they are maintained in the environment and the big task of controlling the scourge. This study detected different AMR genes present in *Salmonella* serovars isolated from chickens and rats collected from 2018 to 2019 in North West province poultry farms in South Africa.

The study detected numerous *β-lactamase* encoded genes (*bla*); *bla_CTX-M_*, *bla_TEM_*, *bla_CTX-M-_*_1)_ in *S. enteritidis* and *S. typhimurium*. However, only *bla_CTX-M_*_-2_ and *bla_CTX-M_*_-15_ genes were detected in *S. typhimurium*. These findings are in agreement with the observations in central Ethiopia by Eguale et al. [42], whereby 79% of *β-lactamase* genes (*bla_TEM_*, *bla_TEM-1_*, *bla_TEM-57_*, *bla_OXA-10_* and *bla_CTX-M-15_*) were detected in animal and human non-typhoidal *Salmonella* isolates. In another study conducted in Egypt, *Salmonella* isolates from chickens were also reported to be harbouring *β-lactamase* resistance genes [43]. However, a previous study in South Africa reported that *Salmonella* isolates haboured *bla*_OXA_, *bla*_CTX-M_, and *bla*_TEM_ from soil and water samples [44]. Our data has shown that 44% of *S. enteritidis* isolates from faecal samples of rats and chickens carried *bla_CTX-M-_*_1_ resistance genes. Various studies from other countries have also reported similar results where *bla_CTX-M-_*_1_ was detected from 100% *Salmonella* isolates obtained from the Senegalese Reference Center for Enterobacteria during 2001–2002 in Senegal [45], from children in Mali [46], from poultry and humans in France and from poultry in Egypt [32]. In general, the current study detected high prevalence of ESBL encoding genes in *Salmonella* isolates. The significance of detecting *β-lactamase* resistance genes raises public health concerns by limiting the therapeutic choices for treating salmonellosis in animals and humans [47], and COL raises major health concerns, as it is used as a treatment of last resort [18,19].

The prevalence of colistin resistance in South Africa from humans was quite low in 2012 (about 3%), but it had climbed significantly to 13 percent by 2014 [48,49]. On April 2016, the South African Medicines Control Council (SAMC) hosted the first meeting of the Colistin Working Group in Pretoria which was aimed at learning more about COL resistance in the country, as well as the value of COL as an antibiotic in humans and animals, and to further start working on a “One Health” strategy [48]. The *mcr*-1 COL resistance gene was first reported from *E. coli* in the Gauteng and Western Cape provinces on samples from livestock and humans [32,50]. Our study has investigated the occurrence of COL resistant genes patterns in *Salmonella* spp., and it has been observed that about 58% and 28% of *S. typhimurium* and *S. enteritidis* isolates were harbouring the *mcr-*4 encoding gene for COL, respectively. A comparable result regarding the prevalence of the *mcr-*4 gene was previously reported in *S. typhimurium* isolates in Italy from pigs [51]. The worrying observation of our study is that the detection frequency of 88% of *mcr-*4 in *S. typhimurium* was from faecal sample isolates of *Rattus* spp. This raises serious concern, as rodents easily adapt to any environment, including human surroundings, and can therefore maintain and distribute the resistance genes in an environment that is difficult to control. This is the first study to detect this gene (*mcr-*4) from *Salmonella* isolated from *Rattus* spp. and chickens in South Africa. Colistin and carbapenems are important antibiotics used to treat MDR bacterial infections in humans [52]. Therefore, the interactions between environment–rat–poultry including humans can encourage the spread of antibiotic-resistant bacteria and resistance genes [53,54]. Rats can get antibiotic resistant bacteria from chicken faeces, as a wide range of antimicrobials are used for chicken growth [55].

Another interesting finding was the presence of *IntI*1 and *IntI*2 encoding genes for integrons with 78% of *S. typhimurium* isolates harbouring *IntI*1, while 49% were carrying *IntI*2. On the other hand, all isolates of *S. enteritidis* were harbouring the *IntI*1 gene while only 33% of *S. enteritidis* isolates from rodents were carrying the *IntI*2 encoding gene. Our data revealed higher prevalence for detection of encoding genes for integrons as compared to a report from Portugal, whereby *IntI*2 was detected from only 3% of *S. typhimurium* isolated from humans, food products, and the environment [56]. The existence of integrons and their flexible transmission have been shown to be ideal for the spread of drug-resistant genes and the acceleration of multidrug resistance [57]. In the matter of multidrug-resistant genes, integrons can encode genes related to the adaptation to different environments [58]. In addition, integrons contain genes that are frequently linked to multidrug resistance [22,58,59].

## 5. Conclusions

The current study revealed a high prevalence of resistance to important antimicrobials such as enrofloxacin, tetracycline, streptomycin, cephalothin, sulphonamide, gentamicin, nalidixic acid, rifampicin, ampicillin and ciprofloxacin. Additionally, this study also found high prevalence of ESBLs in *Salmonella* isolates. The *β-lactamase* encoding genes *bla_CTX-M_, bla_CTX-M-1_, bla_CTX-M-2_, bla_CTX-M-9_, bla_CTX-M-15_, bla_TEM_, bla_SHV_*, and *bla_OXA_* were all detected from *S. enteritidis* and *typhimurium*. Furthermore, the majority of the isolates tested positive for class 1 and 2 integrons, indicating the presence of one or more antibiotic resistance genes. Lastly, the detection of the *mcr*-4 gene in *Salmonella* was a special finding and revealed the extent to which COL resistance is spreading in the country. These findings shed further light on the role of rats as carriers and potential distributors of genes conferring antimicrobial resistance in *Salmonella* from poultry facilities, which could ultimately be transmitted to humans through chicken products. Therefore, it is imperative to control rats at poultry farms in order to reduce the risk of transmission of antibiotic resistance to chickens, and eventually to humans. Future studies are also required to establish the sources of *mcr-*4 and to identify the bacteria that possess the *mcr-*4 gene in South Africa. 

## Figures and Tables

**Table 1:** Antibiotic resistance genes, primers, and PCR conditions used in this study.

Target Gene	Primer	Primer Sequence (5′ → 3′)	Conditions	Cycles	Size (bp)	References
	Colistin resistance					
*mcr-1*	*mcr-*1-F *mcr-*1-R	TATCGCTATGTGCTAAAGCCTG CGTCTGCAGCCACTGGG	94 °C for 5 Min, 94 °C for 30 s, 56 °C for 1 min, 72 °C for 1 min, 72 °C for 5 min.	25	1139	[34]
*mcr-2*	*mcr-*2-F *mcr-*2-R	TATCGCTATGTGCTAAAGCCTG AAAATACTGCGTGGCAGGTAGC			816	
*mcr-*3	*mcr-*3-F *mcr-*3-R	CAATCGTTAGTTACACAATGATGAAG AACACATCTAGCAGGCCCTC			676	
*mcr-*4	*mcr-*4-F *mcr-*4-R	ATCCTGCTGAAGCATTGATG GCGCGCAGTTTCACC			405	
*mcr-*5	*mcr-*5-F *mcr-*5-R	GGTTGAGCGGCTATGAAC GAATGTTGACGTCACTACGG			207	
	*β-lactamase* resistance					
*bla_CTX-M_*	*bla_CTX-M_* -F *bla_CTX-M_*- R	GTTACAATGTGTGAGAAGCAG CCGTTTCCGCTATTACAAAC	94 °C for 5 min, 94 °C for 45 s, 55 °C for 30 s, 72 °C for 60 s, 72 °C for 10 min.	35	550	[35]
*bla_CTX-M-1_*	*bla_CTX-M-_*_1_ -F *bla_CTX-M-_*_1_ -R	GTTACAATGTGTGAGAAGCAG CCGTTTCCGCTATTACAAAC			1041	
*bla_CTX-M-2_*	*bla_CTX-M_*_-2_ -F *bla_CTX-M_*_-2_ -R	ATGATGACTCAGAGCATTCGCCGC TCAGAAACCGTGGGTTACGATTT			876	
*bla_CTX-M-15_*	*bla_CTX-M_*_-15_ -F *bla_CTX-M_*_-15_ -R	CACACGTGGAATTTAGGGACT GCCGTCTAAGGCGATAAACA			995	
*bla_TEM_*	*bla_TEM_* -F *bla_TEM_* -R	TTCTTGAAGACGAAAGGG C ACGCTCAGTGGAACGAAAAC			1150	
*bla_SHV_*	*bla_SHV_* -F *bla_SHV_* -R	CACTCAAGGATGTATTGT G TTAGCGTTGCCAGTGCTCG			885	
*bla_OXA_*	*bla_OXA_*- F *bla_OXA_* -R	ACACAATACATATCAACTTCGC AGTGTGTTTAGAATGGTGATC			813	
	Integrase Class 1, 2 and 3					
*IntI*1	*IntI*1-F *IntI*1-R	GCCTTGCTGTTCTTCTACGG GATGCCTGCTTGTTCTACGG	94 °C for 5 min, 30 s at 94 °C, 30 s, 55–60 °C, 2 min at 72 °C, 5 min at 72 °C.	35	558	[36]
*IntI*2	*IntI*2-F *IntI*2-R	CACGGATATGCGACAAAAAGG TGTAGCAAACGAGTGACGAAATG	94 °C for 5 min, 94 °C for 1 min, 60 °C for 1 min, 72 °C for 2 min, 72 °C for 10 min.	32	740	[25]
*IntI*3	*IntI*3-F *IntI*3-R	GCCTCCGGCAGCGACTTTCAG ACGGATCTGCCAAACCTGACT	94 °C for 10 min, 94 °C for 40 s, 59 °C for 50 s and 72 °C for 55 s 72 °C for 10 min.	30 to 40	650	[37]

**Table 2:** Distribution of antimicrobial resistance from *Salmonella enteritidis* and *typhimurium* isolates.

			No. Resistant (%)		
Antibiotic	Code	Conc. (μg)	*S. typhimurium*	*S. enteritidis*	Total
Ampicillin	AMP	10 μg	3 (6.7%)	−	3 (4.8%)
Sulphonamides	SSS	300 μg	8 (17.8%)	5 (27.8%)	13 (20.6%)
Cephalothin	KF	30 μg	11 (24.4%)	3 (16.7%)	14 (22.2%)
Tetracycline	TE	30 μg	23 (51.1%)	6 (33.3%)	29 (46.0%)
Ciprofloxacin	NA	30 μg	−	2 (11.1%)	2 (3.2%)
Nalidixic acid	C	30 μg	5 (11.1%)	4 (22.2%)	9 (14.3%)
Chloramphenicol	CA	10 μg	−	−	−
Gentamicin	ENR	5 μg	6 (13.3%)	5 (27.8%)	11 (17.5%)
Enrofloxacin	RD	5 μg	26 (57.8%)	13 (72.2%)	39 (61.9%)
Rifampicin	S	10 μg	6 (13.3%)	−	6 (9.5%)
Streptomycin	CIP	5μg	14 (31.1%)	7 (38.9%)	21 (33.3%)

CA = Gentamicin, C = Chloramphenicol, CIP = Ciprofloxacin, RD = Rifampicin, NA = Nalidixic acid, AMP = Ampicillin, ENR = Enrofloxacin, TE = Tetracycline, KF = Cephalothin and SSS = Sulphonamide, S = Streptomycin.

**Table 3:** Detection of different classes of antibiotic resistance genes and integrons from *Salmonella enteritidis* and *typhimurium* isolates.

Serovars	Sample ID	Accession Number	Antimicrobial-Resistant Genes Pattern	Integrase		
				*IntI*1	*IntI*2	*IntI*3
*S*. Typhimurium	R 1	MH352147	*mcr-4*, *bla_CTX-M-2_*, *bla_CTX-M_*_-9_	+	−	−
	R 3	MH352149	*mcr-*4, *bla_CTX-M_*, *bla_CTX-M-2_*, *bla_CTX-M-9_*, *bla_CTX-M-15_*	+	+	−
	R 6	MH352152	*mcr-*4, *bla_CTX-M_*, *bla_CTX-M-2_*, *bla_CTX-M_*_-9_	+	−	−
	R 7	MH352153	*mcr-*4, *bla_CTX-M-2_*, *bla_CTX-M_*_-9_	+	+	−
	R 8	MH352154	*mcr-*4, *bla_CTX-M_*, *bla_CTX-M-2_*, *bla_CTX-M_*_-9_	+	−	−
	R 9	MH352155	*mcr-*4, *bla_CTX-M-2,_bla_CTX-M_*_-9_	+	−	−
	R 10	MH352156	*mcr-*4, *bla_CTX-M-2_*, *bla_CTX-M-9,_ bla_TEM_*	+	+	−
	R 11	MH352157	*bla_CTX-M-2_*, *bla_CTX-M-_*_9_	+	+	−
	R 12	MH352158	*bla_CTX-M-2_*, *bla_CTX-M_*_-9_	+	+	−
	R 22	MH352168	*mcr-*4, *bla_CTX-M_*, *bla_CTX-M_*_-9_	+	+	−
	R 25	MH352171	*mcr-*4, *bla_CTX-M-2_*, *bla_CTX-M_*_-9_	+	+	−
	R 28	MH352174	*mcr-*4, *bla_CTX-M-2_*, *bla_CTX-M-9_*, *bla_TEM_*	+	+	−
	R 29	MH352175	*mcr-*4, *bla_CTX-M_*_-9_	+	+	−
	R 30	MH352176	*mcr-*4, *bla_CTX-M_*_-9_	+	+	−
	R 36	MH352182	*mcr-*4, *bla_CTX-M_*, *bla_CTX-M-9_*, *bla_CTX-M_*_-15_	+	+	−
	R 37	MH352183	*mcr-*4, *bla_CTX-M_*_-9_	+	−	−
	R 39	MH352185	*mcr-*4, *bla_CTX-M-2_*, *bla_CTX-M_*_-9_	+	−	−
	R 43	MH352189	*mcr-*4, *bla_CTX-M-2_*, *bla_CTX-M-9_*, *bla_TEM_*	+	+	−
	R 44	MH352190	*mcr-*4, *bla_CTX-M_*_-9_	+	−	−
	R 45	MH352191	*mcr-*4, *bla_CTX-M_*_-9_	+	+	−
	R 46	MH352192	*mcr-*4, *bla_CTX-M_*_-9_	+	−	−
	R 48	MH352194	*bla_CTX-M-2_*, *bla_CTX-M_*_-9_	+	−	−
	R 49	MH352195	*bla_CTX-M-2_*, *bla_CTX-M-9_*, *bla_TEM_*	+	+	−
	R 51	MH352197	*bla_CTX-M-2_*, *bla_CTX-M-9_*	+	−	−
	R 52	MH352198	*bla_CTX-M-2_*, *bla_CTX-M-9_*	+	−	−
	R 53	MH352199	*mcr-*4, *bla_CTX-M_*, *bla_CTX-M-2_*, *bla_CTX-M_*_-15_	+	+	−
	R 54	MH352200	*mcr-*4, *bla_CTX-M-2_*, *bla_CTX-M_*_-9_	+	−	−
	R 56	MH352202	*bla_CTX-M-2_*, *bla_CTX-M-9_*	+	−	−
	R 60	MH352206	*bla_CTX-M-9_*	+	+	−
	R 65	MH352211	*mcr-*4, *bla_CTX-M_*_-9_	+	+	−
	R 67	MH352213	*mcr-*4, *bla_CTX-M-2_*, *bla_CTX-M-9_*, *bla_TEM_*	+	−	−
	C 6	MH356675	*mcr-*4	−	−	−
	C 7	MH356676	*mcr-*4, *bla_CTX-M_*	+	+	−
	C 11	MH356680	*mcr-*4, *bla_CTX-M_*, *bla_CTX-M_*_-15_	−	−	−
	C 12	MH356681	−	−	+	−
	C 23	MH356692	−	+	−	−
	C 26	MH356695	*bla_CTX-M-15_*	−	−	−
	C 28	MH356697	*bla_CTX-M_*, *bla_CTX-M-15_*	−	+	−
	C 30	MH356699	*bla_CTX-M-15_*	−	−	−
	C 32	MH356701	−	−	−	−
	C 34	MH356703	−	−	−	−
	C 34	MH356704	−	−	−	−
	C 36	MH356705	−	−	+	−
	C 37	MH356706	−	−	−	−
	C 41	MH356710	*bla_CTX-M-15_*	+	−	−
	C 42	MH356711	−	−	−	−
	C 43	MH356712	*bla_CTX-M_*	−	−	−
	C 44	MH356713	−	−	−	−
	C 45	MH356714	−	+	+	−
	C 46	MH356715	*bla_CTX-M_*_-15_	−	−	−
*S*. Enteritidis	C 1	MH356670	*bla_CTX-M_*_-15_	+	−	−
	C 8	MH356677	*mcr-*4	+	−	−
	C 20	MH356689	−	+	+	−
	C 22	MH356691	−	+	−	−
	C 29	MH356698	*mcr-*4	+	−	−
	C 40	MH356709	*mcr-*4, *bla_CTX-M-1_*	+	−	−
	R 2	MH352148	*mcr-*4, *bla_CTX-M-_*_1_	+	−	−
	R 4	MH352150	*bla_CTX-M-1_*	+	−	−
	R 7	MH352153	*bla_CTX-M-1_*	+	+	−
	R 27	MH352173	*bla_CTX-M_*	+	−	−
	R 37	MH352183	*bla_CTX-M_*, *bla_CTX-M-15_*	+	−	−
	R 50	MH352196	*bla_CTX-M_*, *bla_SHV_*	+	+	−
	R 57	MH352203	*mcr-*4, *bla_CTX-M_*, *bla_SHV_*	+	+	−
	R 58	MH352204	*bla_CTX-M_*, *bla_CTX-M-1_*, *bla_CTX-M-15_*, *bla_SHV_*	+	−	−
	R 59	MH352205	*bla_CTX-M_*, *bla_CTX-M-15_*	+	−	−
	R 62	MH352208	*bla_CTX-M_*, *bla_CTX-M-1_*, *bla_CTX-M-15,_bla_SHV_*	+	−	−
	R 64	MH352210	*bla_CTX-M-1_*, *bla_CTX-M-15_*	+	+	−
	R 68	MH352214	*bla_CTX-M_*, *bla_CTX-M-1_*	+	+	−

## Data Availability

The data presented in this study are available on request from the corresponding author.

## Supplementary Materials

The following are available online at https://www.mdpi.com/article/10.3390/microorganisms10020313/s1, Figure S1: Representative agarose gel image of the *bla_CTX-M_* gene products. Lane M: 100 bp DNA ladder; Lanes 2–11 positive gene fragments and Lane 1: negative control. Figure S2: Representative agarose gel image of the *bla_CTX-M-_*_1_ gene products. Lane M: 100 bp DNA ladder; Lanes 2–11 positive gene fragments and Lane 1: negative control. Figure S3: Representative agarose gel image of the *bla_CTX-M-_*_2_ gene products. Lane M: 100 bp DNA ladder; Lanes 1, 3–6, 9 positive gene fragments and Lane 11: negative control. Figure S4: Representative agarose gel image of the *bla_CTX-M-_*_15_ gene products. Lane M: 100 bp DNA ladder; Lanes 2–17 positive gene fragments and Lane 1: negative control. Figure S5: Representative agarose gel image of the *bla_OXA_* gene products. Lane M: 100 bp DNA ladder; Lane 2 positive gene fragments and Lane 1: negative control. Figure S6: Representative agarose gel image of the *bla_TEM_* gene products. Lane M: 100 bp DNA ladder; Lanes 2–11 positive gene fragments and Lane 1: negative control. Figure S7: Representative agarose gel image of the *bla_SHV_* gene products. Lane M: 100 bp DNA ladder; Lanes 3–5, 8–11 positive gene fragments and Lane 1: negative control. Figure S8: Representative agarose gel image of the *IntI*1 gene products. Lane M: 100 bp DNA ladder; Lanes 1-8 positive gene fragments and Lane 1: negative control. Figure S9: Representative agarose gel image of the *IntI*2 gene products. Lane M: 100 bp DNA ladder; Lanes 1-8 positive gene fragments and Lane 1: negative control. Figure S10: Representative agarose gel image of the *mcr-*4 gene products. Lane M: 100 bp DNA ladder; Lanes 1–8 positive gene fragments and Lane 1: negative control.
