# Supplementary material for: Molecular Detection of Integrons, Colistin and β-lactamase Resistant Genes in Salmonella enterica Serovars Enteritidis and Typhimurium Isolated from Chickens and Rats Inhabiting Poultry Farms
Source: Microorganisms. 2022 Jan 28;10(2):313. doi: 10.3390/microorganisms10020313 (PMC8876313; doi:10.3390/microorganisms10020313)
Supplement: Supplementary file 1 [file microorganisms-10-00313-s001.zip › microorganisms-1496093-supplementary.pdf]

Supplementary Materials

Molecular detection of integrons, colistin and *β-lactamase* resistant genes in *Salmonella enterica* serovars Enteritidis and Typhimurium isolated from chickens and rats inhabiting poultry farms

Tsepo Ramatla [1,2,*], Kealeboga Mileng [1], Rendani Ndou [1], Nthabiseng Mphuti [1], Michelo Syakalima [1,3], Kgaugelo E. Lekota [2] and Oriel M.M. Thekisoe [2]

[1] Department of Animal Health, School of Agriculture, North-West University, Private Bag X2046, Mmabatho 2735, South Africa; k.mileng@gmail.com (K.M.); Rendani.Ndou@nwu.ac.za (R.N.); nthabiseng.mphuthi@nwu.ac.za (N.M.); michsan65@gmail.com (M.S.)

[2] Unit for Environmental Sciences and Management, North-West University, Private Bag X6001, Potchefstroom 2531, South Africa; Lekota.Lekota@nwu.ac.za (K.E.L.); thekisoe@gmail.com (O.M.M.T.)

[3] Department of Disease Control, School of Veterinary Medicine, University of Zambia, Lusaka P.O. Box 32379, Zambia

* Correspondence: ra21205450@gmail.com; Tel.: +27-18-299-2521

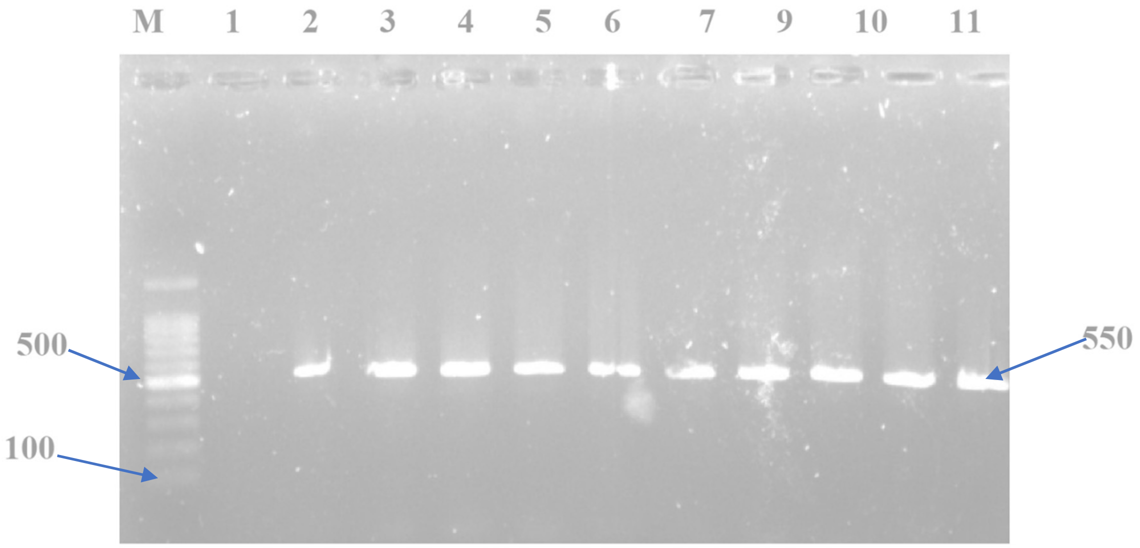

Figure S1. Representative agarose gel image of the *bla*$_{CTX-M}$ gene products. Lane M: 100bp DNA ladder; Lanes 2-11 positive gene fragments and Lane 1: negative control.

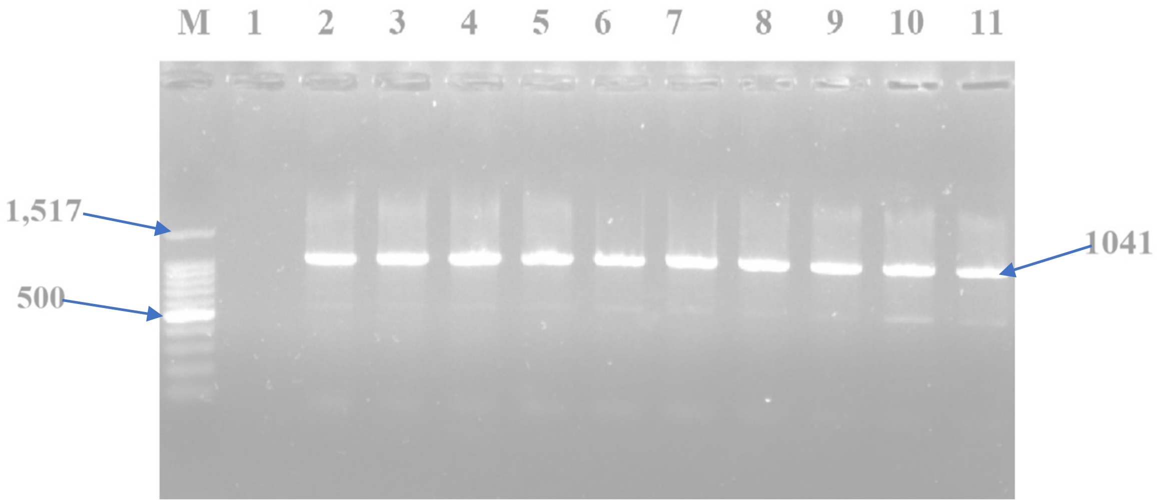

Figure S2. Representative agarose gel image of the *bla*$_{CTX-M-1}$ gene products. Lane M: 100bp DNA ladder; Lanes 2-11 positive gene fragments and Lane 1: negative control.

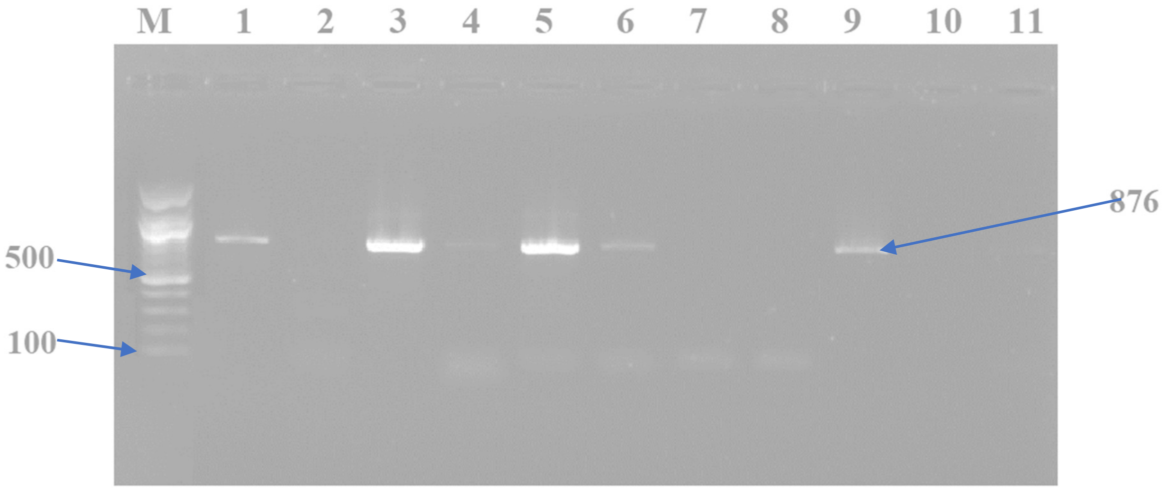

Figure S3. Representative agarose gel image of the *bla*$_{CTX-M-2}$ gene products. Lane M: 100bp DNA ladder; Lanes 1, 3-6, 9 positive gene fragments and Lane 11: negative control.

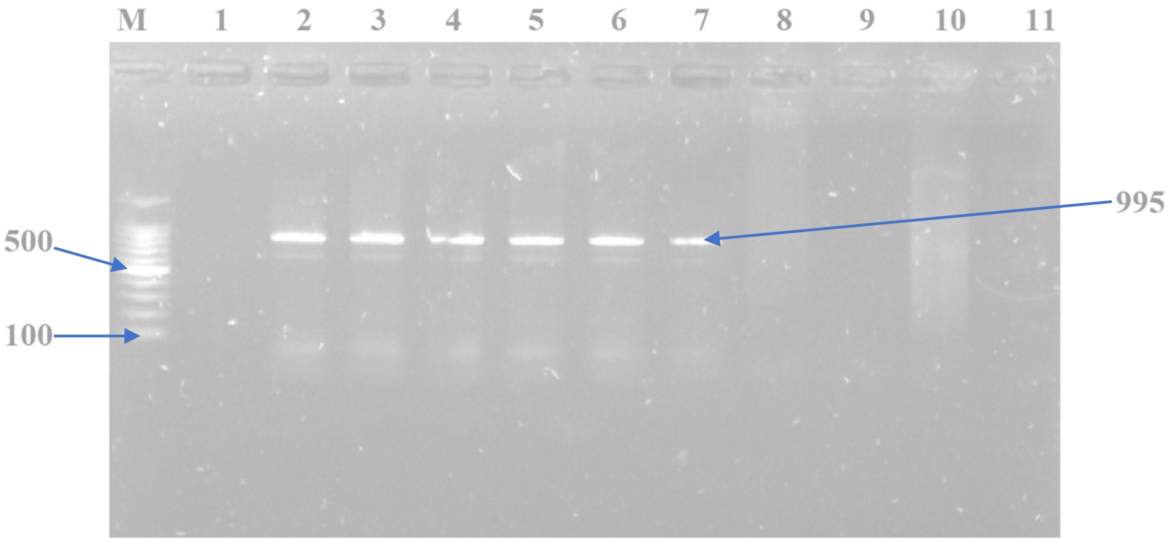

Figure S4. Representative agarose gel image of the *bla*$_{CTX-M-15}$ gene products. Lane M: 100bp DNA ladder; Lanes 2-17 positive gene fragments and Lane 1: negative control.

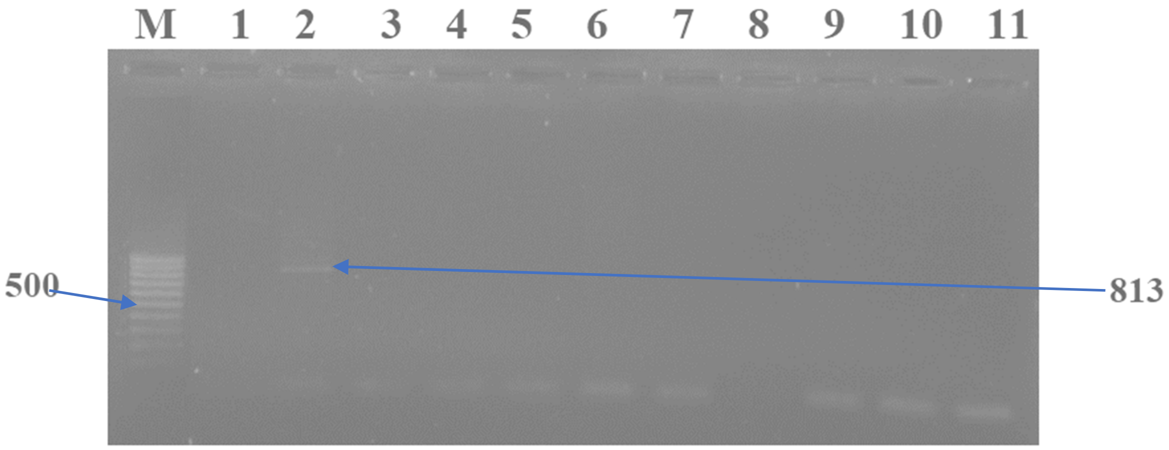

Figure S5. Representative agarose gel image of the *bla*$_{OXA}$ gene products. Lane M: 100bp DNA ladder; Lane 2positive gene fragments and Lane 1: negative control.

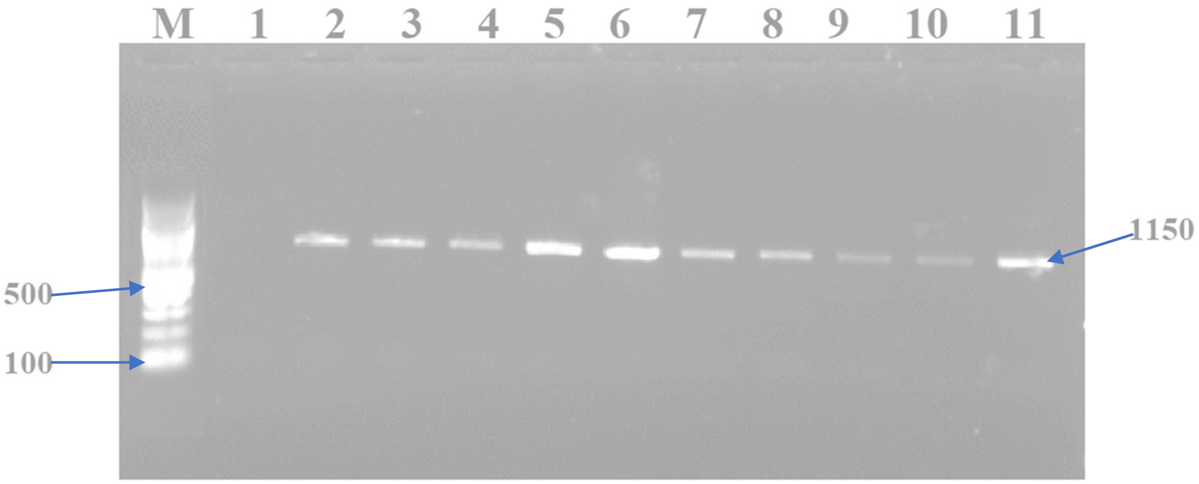

Figure S6. Representative agarose gel image of the *bla*$_{TEM}$ gene products. Lane M: 100bp DNA ladder; Lanes 2-11 positive gene fragments and Lane 1: negative control.

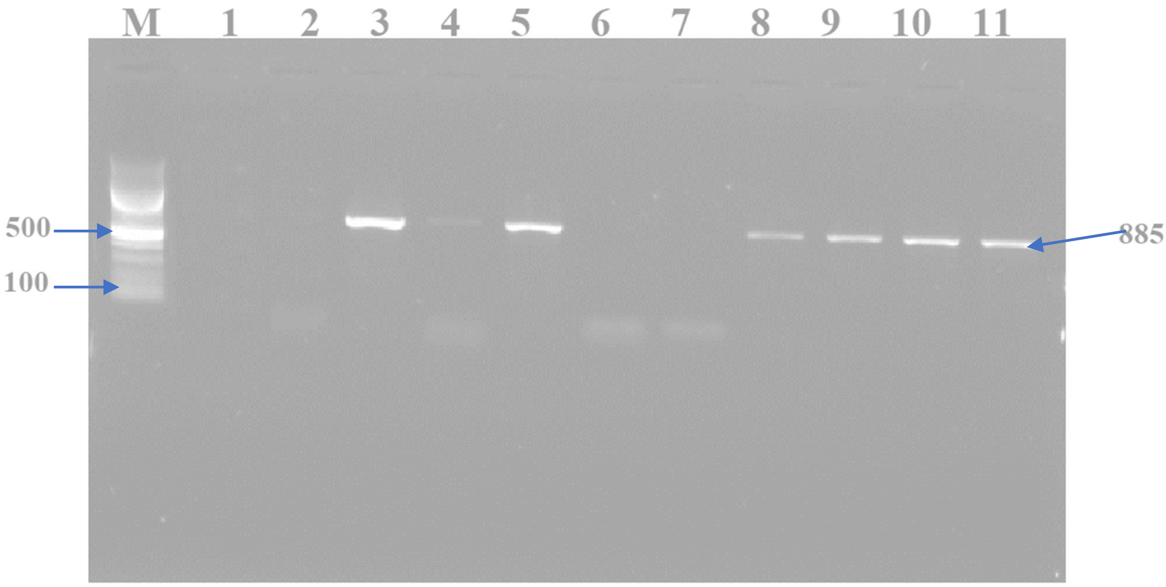

Figure S7. Representative agarose gel image of the *bla*$_{SHV}$ gene products. Lane M: 100bp DNA ladder; Lanes 3-5, 8-11 positive gene fragments and Lane 1: negative control.

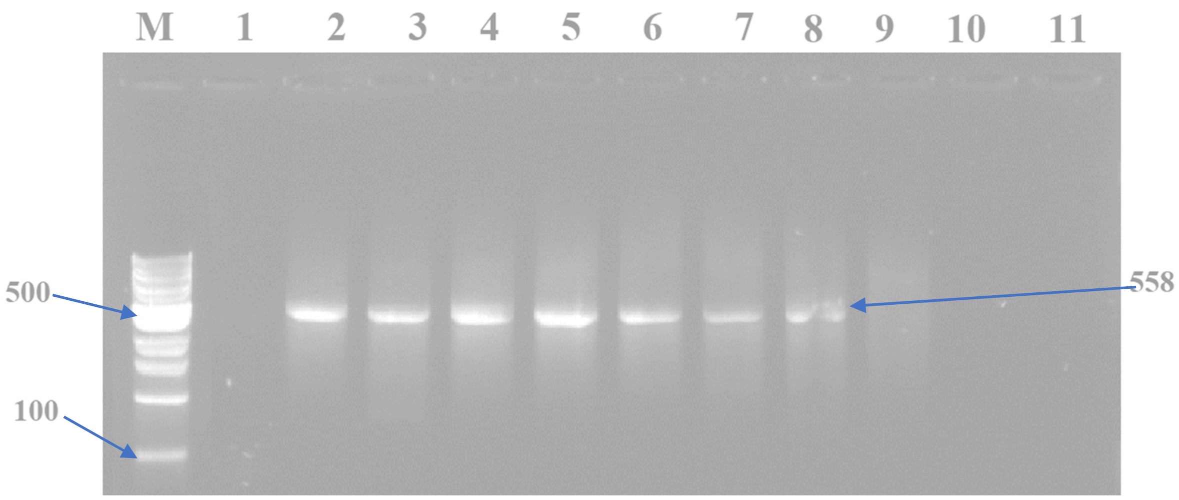

Figure S8. Representative agarose gel image of the *IntI*1 gene products. Lane M: 100bp DNA ladder; Lanes 1-8 positive gene fragments and Lane 1: negative control.

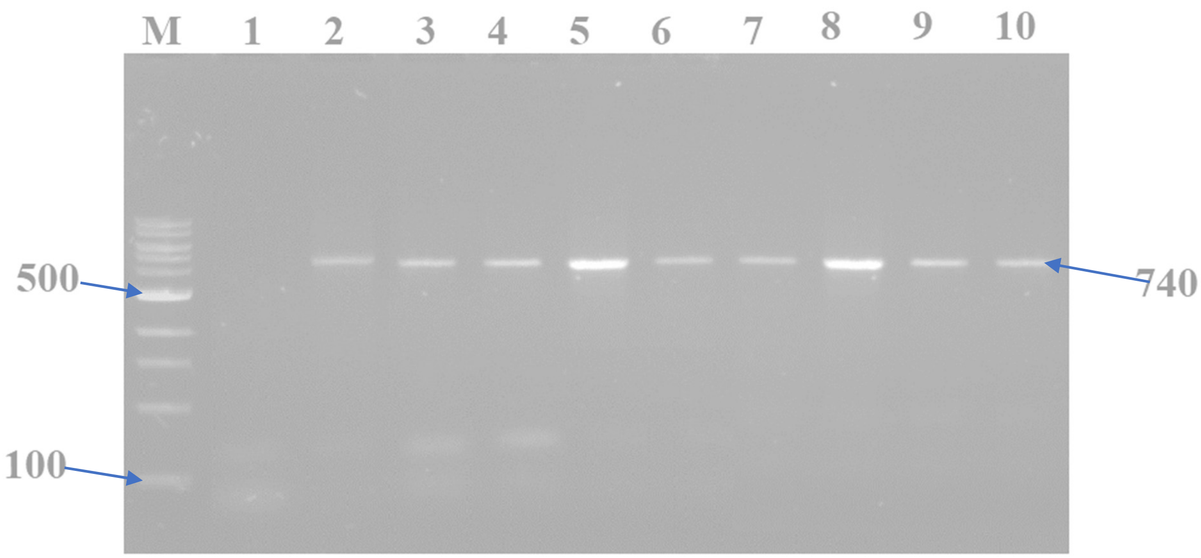

Figure S9. Representative agarose gel image of the *IntI*2 gene products. Lane M: 100bp DNA ladder; Lanes 1-8 positive gene fragments and Lane 1: negative control.

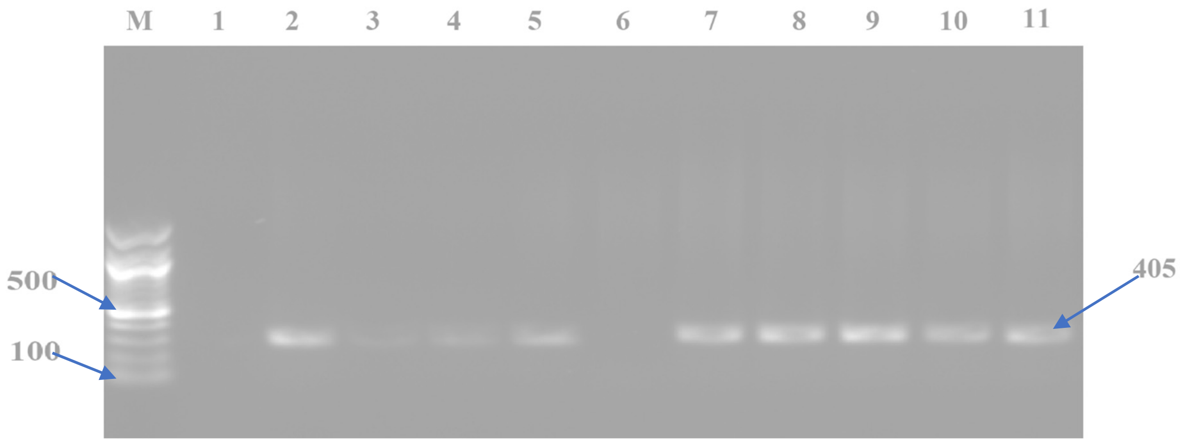

Figure S10. Representative agarose gel image of the *mcr*-4gene products. Lane M: 100bp DNA ladder; Lanes 1-8 positive gene fragments and Lane 1: negative control.
